# Case report: Spontaneous arterial bleeding in the lateral thoracic region during helmet CPAP treatment: a report of three cases in patients with severe COVID-19

**DOI:** 10.3389/fmed.2024.1418029

**Published:** 2024-11-29

**Authors:** Vincenzo Francesco Tripodi, Salvatore Sardo, Salvatore Silipigni, Alberto Stagno, Antonio Francesco Neri, Antonio Bottari, Anna Teresa Mazzeo

**Affiliations:** ^1^Unit of Anaesthesia and Intensive Care, Department of Human Pathology in Adulthood and Childhood, University of Messina, Messina, Italy; ^2^Department of Medical Sciences and Public Health, Faculty of Medicine and Surgery, University of Cagliari, Cagliari, Italy; ^3^Section of Radiological Sciences, Department of Biomedical, Dental and Morphological and Functional Imaging Sciences, University of Messina, Messina, Italy

**Keywords:** COVID-19, SARS-CoV-2, continuous positive airway pressure, ARDS, respiratory failure, embolization

## Abstract

Severe acute respiratory syndrome coronavirus 2 (SARS-CoV-2) infection has put enormous pressure on healthcare systems worldwide. While the majority of severe cases present with respiratory failure, thrombosis or bleeding have also been reported at unusual sites. Major bleeding, particularly in patients treated with therapeutic anticoagulation, has been observed between the second and third week after the onset of SARS-CoV-2 infection. This article describes three cases of patients admitted to the hospital with severe SARS-CoV-2 pneumonia who had spontaneous arterial bleeding from the thoracic and subscapular regions during treatment with helmet continuous positive airway pressure (H-CPAP) in the intensive care unit (ICU), requiring a percutaneous embolization procedure. A possible correlation with helmet-supported ventilation is hypothesized.

## Introduction

Severe acute respiratory syndrome coronavirus 2 (SARS-CoV-2) is a coronavirus strain that caused the coronavirus disease 2019 (COVID-19) pandemic (1, 2).

During SARS-CoV-2 infection, the virus primarily targets the lungs, causing severe inflammation and damage to the alveoli, leading to respiratory distress. This can develop into pneumonia, acute respiratory distress syndrome (ARDS), and, in severe cases, lung failure. In addition, SARS-CoV-2 triggers abnormal blood clotting, which increases the risk of thrombosis. Microthrombi can form in the blood vessels of the lungs, further impairing oxygen exchange and contributing to the severity of the disease. These effects on both the lungs and the coagulation system are key features of severe COVID-19 cases (3).

Patient management primarily focuses on supportive care, which includes oxygenation, fluid management, and treatment with various drugs, such as antiviral therapies, antibiotics, steroids, non-steroidal anti-inflammatory drugs, and immunosuppressive drugs. Patients with COVID-19 pneumonia may develop acute hypoxemic respiratory failure (AHRF) and require positive end-expiratory pressure (PEEP) (2–4).

COVID-19 patients have also shown alterations in coagulation tests, with a significant increase in D-dimer levels associated with the severity of illness and adverse outcomes (4). In addition, a high risk of venous thromboembolism has been identified, with a high prevalence of symptomatic acute pulmonary embolism and deep vein thrombosis (4).

In patients requiring respiratory support, non-invasive ventilation (NIV) administered through a helmet has been widely used, even for prolonged periods. This approach reduces the need for intubation, although its effect on outcomes can vary (1–4). The helmet is an alternative interface for delivering NIV, consisting of a clear plastic hood mounted on a hard-plastic ring with a multi-size silicon-polyvinyl chloride (PVC) soft collar to fit a wide range of neck dimensions and to surround the entire head of the patient (1–4).

The World Health Organization conducted a systematic review of ventilation strategies for coronaviruses, including MERS, SARS, and COVID-19, and concluded that while NIV may reduce mortality and the need for intubation, it also has the potential to increase the risk of transmission to healthcare workers (3).

Traditional fixation systems used to anchor the helmet during CPAP can worsen the discomfort or pain experienced by patients and cause device-related pressure ulcers (2, 3). The most commonly used solutions for securing helmet non-invasive ventilation (H-NIV) setups are armpit straps and counterweight systems (5).

In a recent meta-analysis of trials involving adults with AHRF, treatment with non-invasive respiratory support, including H-NIV, was associated with a lower risk of death compared with standard oxygen therapy (6). H-NIV reduces air leaks compared to face mask interfaces, potentially decreasing viral transmission when used to treat patients with AHRF caused by COVID-19 (6, 7).

In various forms of AHRF, H-NIV may enhance the recruitment of non-aerated alveoli in dependent pulmonary regions, thereby increasing lung functional residual capacity and decreasing shunting (2–4).

The most commonly used solutions for fixing H-NIV setups are axillary straps and counterweight systems. However, this traditional securing system may compress the lateral thoracic wall and subscapular region, worsening the pain and discomfort experienced by the patient and causing device-related local complications, such as hematomas, edema, and pressure ulcers (6–8).

## Case presentation

We describe three cases of adult patients admitted to the intensive care unit (ICU) of the University Hospital “Gaetano Martino” of Messina between 2021 and 2022. These patients were diagnosed with COVID-19 pneumonia and required helmet continuous positive airway pressure (H-CPAP). They also developed spontaneous arterial bleeding in the thoracic region, necessitating embolization. During hospitalization, the patients were treated with enoxaparin and initially received high-flow nasal cannula (HFNC) oxygen. As their respiratory failure worsened, H-NIV was initiated. Within the first 48–96 h from the start of the H-NIV treatment, arterial bleeding from vessels located in the area compressed by the helmet straps was documented.

**Figure 1:**
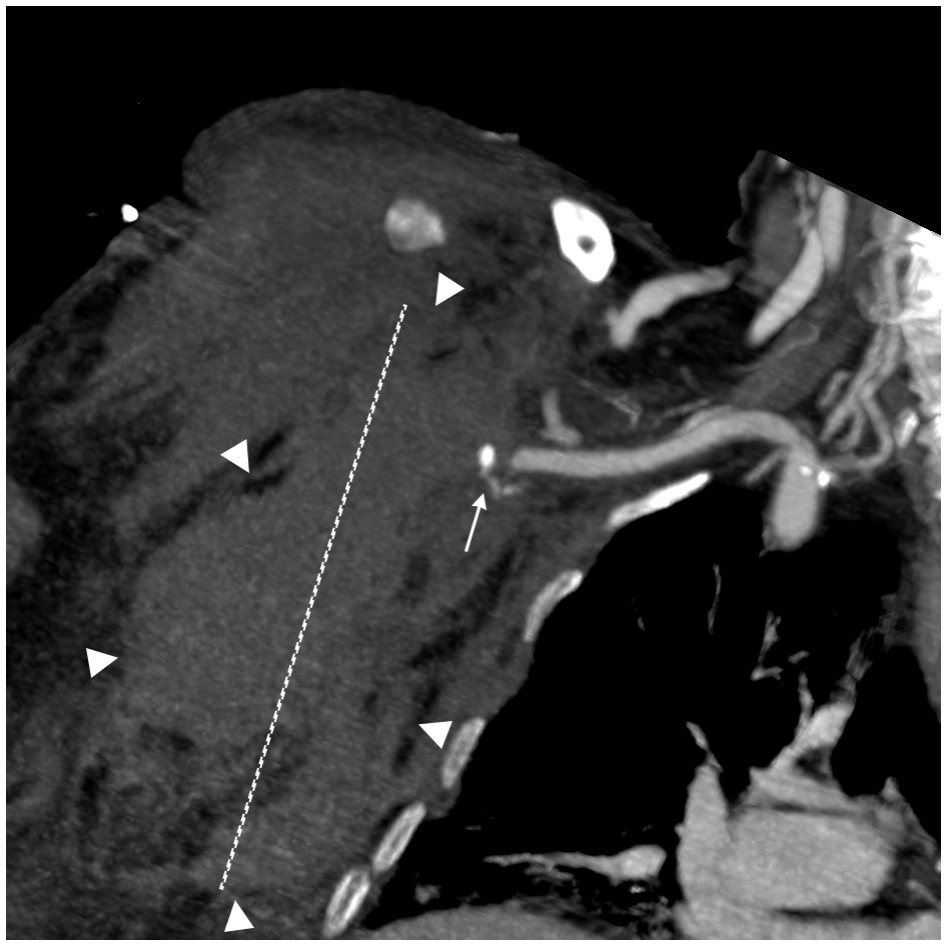
Spontaneous bleeding in the right pectoral region. A coronal Multiplanar reconstruction (MPR) CT angiography image; arterial phase. A small pseudoaneurysm (white arrow) arising from a minor branch of the subscapular artery is visible near the subclavian artery. A large hematoma in the right pectoral and thoracic regions (maximum diameter 10 cm—white dashed line).

The first patient was an 82-year-old woman weighing 62 kg with a BMI of 21.97. Her clinical history included arterial hypertension, chronic ischemic heart disease, diabetes mellitus, and pulmonary emphysema. She was admitted for bilateral interstitial pneumonia due to SARS-CoV-2, complicated by pulmonary embolism. The patient received 8,000 UI of enoxaparin twice a day. She experienced spontaneous bleeding in the right pectoral and subscapular regions 72 h after the start of the treatment. Figure 1 shows a large hematoma in the right pectoral region measuring more than 10 cm. She underwent successful embolization using detachable coils and n-butyl cyanoacrylate glue. At the time of the onset of bleeding, her platelet count was 197.000 /mmc. She died of septic shock 39 days after being admitted to the ICU (Table 1).

**Table 1:** Summary of the clinical characteristics, laboratory values, management, and outcomes of the three patients.

**Variable**	**Case 1**	**Case 2**	**Case 3**
Age	82	78	79
Sex	Female	Male	Male
Weight	62 kg	78 kg	74 kg
BMI	21.97	26.6	27.18
Medical history	Arterial hypertension, chronic ischemic heart disease, diabetes mellitus, and pulmonary emphysema	Chronic obstructive pulmonary disease, arterial hypertension, carotid vasculopathy, and hypercholesterolemia	Arterial hypertension, diabetes mellitus, peripheral vascular disease, and prostate cancer
COVID-19 diagnostic test RT-PCR	Yes	Yes	Yes
Pneumonia	Yes	Yes	Yes
H-FNC during hospitalization	Yes	Yes	Yes
Intubated during hospitalization	Yes	No	Yes
Parameters at the time of H-NIV treatment initiation	FiO_2_ 60%	FiO_2_ 50%	FiO_2_ 60%
	PEEP 6	PEEP 6	PEEP 8
	Pressure support (Ps) 6	Pressure support (Ps) 8	Pressure support (Ps) 10
Sedation during H-NIV	Dexmedetomidine	Dexmedetomidine	Dexmedetomidine
	0.6–1.2 mcg/kg/h iv	0.4–0.8 mcg/kg/h iv	0.6–1.2 mcg/kg/h iv
Day of hospitalization (after admission to the hospital) at the onset of bleeding	8	24	6
Size of the hematoma	10 cm	13 cm	12 cm
**Laboratory values at the time of admission to the ICU**			
WBC (K/mm^3^)	20.600	13.000	10.600
Hemoglobin (g/dl)/hematocrit (%)	10.1/30	11.4/32	9.8/26
Platelets (K/mm^3^)	223.000	378.000	219.000
Fibrinogen (mg/dl)	527	450	592
Creatinine (mg/dl)	0.6	0.9	1.1
**Laboratory values at the time of the bleeding episode**			
WBC (K/mm^3^)	16.400	13.000	23.000
Hemoglobin (g/dl)/Hematocrit (%)	7.1/22	7.7/23	6.3/17
Platelets (K/mm^3^)	197.000	224.000	191.000
Fibrinogen (mg/dl)	601	590	810
Creatinine (mg/dl)	0.9	1.2	1.6
Site of bleeding/size of the hematoma	Right pectoral and subscapular regions	Right thoracic and humeral regions	Subscapular artery
**Other data**			
Prior history of bleeding	No	No	No
Trauma	No	No	No
CRRT	No	No	Yes
Vasopressors iv	Yes	No	Yes
Hospital length of stay	39	34	34
ICU length of stay	32	10	34
Outcome	Deceased	Discharged	Deceased

**Figure 2:**
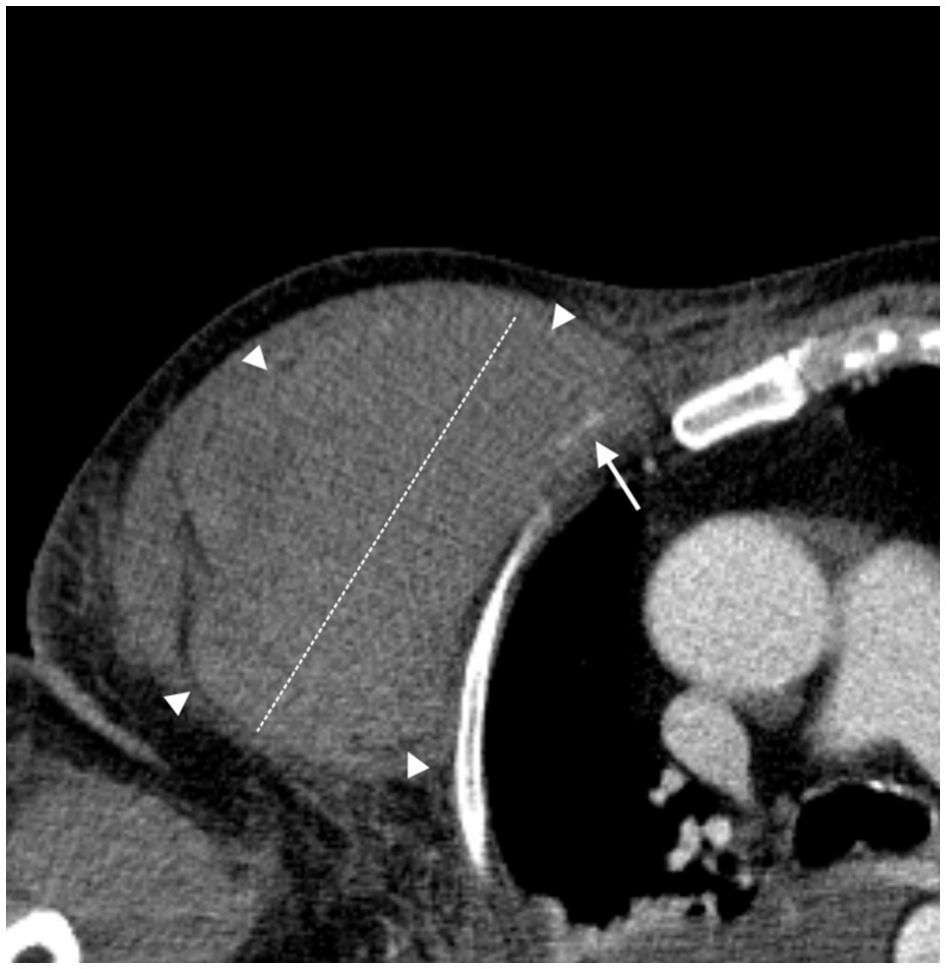
Spontaneous bleeding in the right thoracic and humeral region. Axial CT angiography image; arterial phase. A slightly hyperdense intramuscular pectoral hematoma (diameter 13 cm—white dashed line) due to recent bleeding (borders are marked with white arrowheads). The CT angiography shows active bleeding as a slight linear hyperdensity (white arrow) of the fourth anterior intercostal artery, a branch of the internal mammary artery.

The second patient was a 78-year-old man weighing 78 kg with a BMI of 26.6. He had a history of chronic obstructive pulmonary disease, arterial hypertension, carotid vasculopathy, and hypercholesterolemia and was admitted for SARS-CoV-2 bilateral interstitial pneumonia and sepsis. He received 4,000 UI of enoxaparin twice a day, and his platelet count was 224.000/mmc. After 3 days in the ICU and 2 days of H-NIV treatment (Table 1), the patient developed hemorrhagic shock, severe hypotension, and anemia caused by spontaneous bleeding in the right thoracic and humeral regions. Figure 2 shows active bleeding (white arrow) from the fourth anterior intercostal artery, a branch of the internal mammary artery, and a 13-cm hematoma. After 34 days of hospitalization, he was discharged to a respiratory rehabilitation facility and is currently in good health.

The third patient was a 79-year-old man weighing 74 kg, with a BMI of 27.18. He was admitted to the ICU from the pneumology department. He was diagnosed with SARS-CoV-2 ARDS and sepsis, and he had a clinical history of arterial hypertension, diabetes mellitus, peripheral vascular disease, and prostate cancer. The patient received 4,000 UI of enoxaparin twice a day and was treated with H-NIV using a helmet from the time of admission to the ICU. On day 2, anuria and severe anemia (hemoglobin 6.3 g/dl; hematocrit 18%; platelets 191.000/mmc) prompted an emergency CT scan, which revealed spontaneous bleeding from the subscapular artery. The bleeding was diagnosed 48 h after starting the treatment (Table 1).

**Figure 3:**
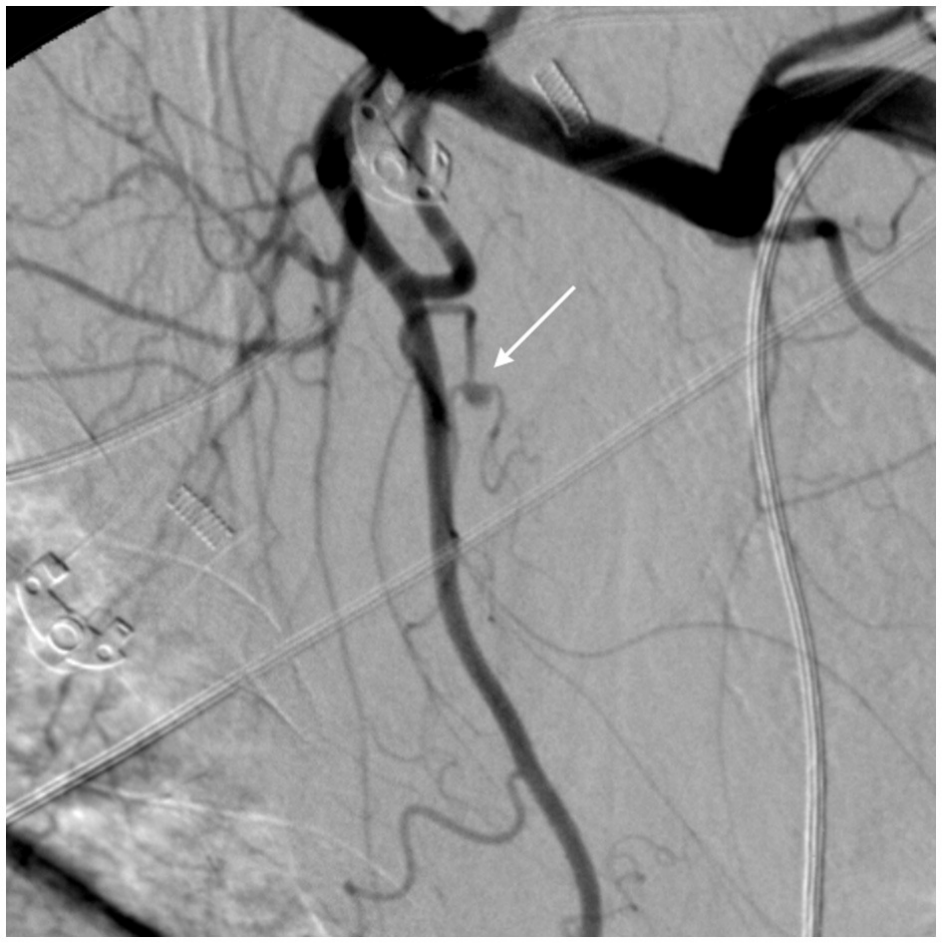
Large left pectoral hematoma due to spontaneous bleeding—digital subtraction angiography (DSA) frame. The DSA shows a 3 mm pseudoaneurysm (white arrow) arising from a small branch of the subscapular artery. The pseudoaneurysm represents one of the most common signs of vascular damage and requires embolization.

Figure 3 shows spontaneous bleeding caused by a pseudoaneurysm in a small branch of the subscapular artery. The patient underwent emergency angiography and successful embolization using detachable microcoils. The patient was mechanically ventilated for 21 days and received continuous renal replacement therapy (CRRT) using continuous veno-venous hemofiltration with dialysis filtration (CVVHDF) for 6 days after the acute bleeding problem was resolved. He died 34 days after ICU admission due to complications from acute respiratory distress syndrome (ARDS) and acute kidney injury (AKI).

## Discussion

Helmet non-invasive ventilation (H-NIV) has been used in patients with COVID-19 based on the assumption that the helmet interface is more effective than the mask interface for delivering prolonged treatments with high positive airway pressure. However, data on its effectiveness are limited (7).

In a randomized controlled trial (RCT) involving 322 patients, Arabi et al. compared the use of H-NIV with standard respiratory support. The primary outcome was 28-day all-cause mortality. The results of this study suggested that H-NIV did not significantly reduce 28-day mortality compared with usual respiratory support in patients with acute hypoxemic respiratory failure caused by COVID-19 pneumonia (7).

In a systematic review and meta-analysis that included 16 RCTs and eight observational studies, Chauduri et al. examined the effect of H-NIV compared to facemask NIV or high-flow nasal cannula (HFNC) in cases of acute respiratory failure (9). The authors concluded that H-NIV, compared to facemask NIV, may reduce mortality and intubation; however, the effect of H-NIV compared to HFNC remains uncertain (9).

While AHRF is the main manifestation of severe COVID-19, the most appropriate form of respiratory support and the optimal timing remain to be defined. A well-seated helmet is generally better tolerated than an oronasal or full-face mask, especially for extended CPAP therapy over several days (2, 6). Tolerance to non-invasive support, ability to clear secretions, worsening of lung injury from large tidal volumes during inspiratory pressure support, and self-induced lung injury are among the main concerns associated with NIV. Additionally, potential harm resulting from skin breakdown, arm edema, or thrombosis has also been reported (6).

In a multicenter randomized clinical trial conducted in four intensive care units (ICUs) in Italy between October and December 2020, 109 patients with moderate to severe hypoxemic respiratory failure caused by COVID-19 were randomly assigned to receive continuous treatment with H-NIV for at least 48 h or high-flow oxygen alone (10). The primary outcome was the number of days free from respiratory support within 28 days after enrollment. The study showed that there were no significant differences between the two groups of patients in terms of the number of days free from respiratory support within 28 days (10).

We have described three cases of acute severe bleeding in the lateral thoracic and subscapular regions, associated with the straps of the device used, in patients admitted to the ICU for severe HARF due to COVID-19 treated with H-NIV.

The risk of bleeding in coronavirus disease is primarily related to thromboembolic or vascular fragility complications that may develop. Therefore, managing the balance between thrombosis prevention and bleeding risk is crucial for effective treatment.

An axillary hematoma is also dangerous because it can lead to neurological deficits. The brachial plexus within the axillary sheath contains the median, ulnar, radial, and musculocutaneous nerves. Therefore, any compression must be treated promptly. Direct compression of a nerve can cause demyelination of axons, resulting in a transient conduction block. Prolonged compression can lead to Wallerian degeneration, resulting in motor nerve impairment (11). The hypothesis is that the straps of the device used have obstructed vascular flow (venous and arterial), progressively resulting in extravasation from the vessel walls, despite preventive measures. One possibility is that increased intrathoracic pressure may have caused hypertension in the scapular and pectoral arteries, which could have consequently promoted bleeding. From another perspective, it is possible that traumatic damage to the vessels occurred during the placement or removal of the device in the ICU. Therapeutic anticoagulation for COVID-19, aimed at addressing local microvascular and macrovascular thrombosis, may have also contributed to increased bleeding (6).

Previous extensive studies, including those by Arabi et al. (7), Colaianni-Alfonso et al. (8), and Al Hashim et al. (12), reported no association between H-CPAP and H-NIV and lateral chest wall bleeding.

Significant bleeding at unusual sites can occur in COVID-19 patients undergoing anticoagulation treatment, but a possible correlation with H-NIV has not been previously reported (7, 13, 14). It is difficult to demonstrate a correlation between the bleeding experienced and the use of the device; however, the impact and decubitus of devices used in bedridden patients, and the importance of nursing procedures in the ICU, should not be underestimated.

## Data Availability

The original contributions presented in the study are included in the article/supplementary material, further inquiries can be directed to the corresponding author.
